# Concurrent malaria and arbovirus infections in Kedougou, southeastern Senegal

**DOI:** 10.1186/s12936-016-1100-5

**Published:** 2016-01-28

**Authors:** Abdourahmane Sow, Cheikh Loucoubar, Diawo Diallo, Oumar Faye, Youssoupha Ndiaye, Cheikh Saadibou Senghor, Anta Tal Dia, Ousmane Faye, Scott C. Weaver, Mawlouth Diallo, Denis Malvy, Amadou Alpha Sall

**Affiliations:** Arbovirus and Viral Hemorrhagic Fevers Unit, Institut Pasteur Dakar, 36 Avenue Pasteur, BP 220 Dakar, Senegal; Medical Entomology Unit, Institut Pasteur Dakar, 36 Avenue Pasteur, BP 220 Dakar, Senegal; Saraya Health District, Saraya, Senegal; Kedougou Health District, Kedougou, Senegal; Institut Santé et développement (ISED), Université Cheikh Anta Diop, Dakar, Senegal; Department of Pathology, Institute for Human Infections and Immunity, Center for Tropical Diseases, University of Texas Medical Branch, Galveston, TX USA; Institut de Santé Publique d’Epidémiologie et de Développement (ISPED), Centre de recherche INSERM U897 Epidémiologie-Biostatistique, Université de Bordeaux, Bordeaux, France

**Keywords:** Arbovirus, Malaria, Co-infection, Kedougou

## Abstract

**Background:**

Malaria is one of the leading causes of acute febrile illness (AFI) in Africa. With the advent of malaria rapid diagnostic tests, misdiagnosis and co-morbidity with other diseases has been highlighted by an increasing number of studies. Although arboviral infections and malaria are both vector-borne diseases and often have an overlapping geographic distribution in sub-Saharan Africa, information about their incidence rates and concurrent infections is scarce.

**Methods:**

From July 2009 to March 2013 patients from seven healthcare facilities of the Kedougou region presenting with AFI were enrolled and tested for malaria and arboviral infections, i.e., yellow fever (YFV), West Nile (WNV), dengue (DENV), chikungunya (CHIKV), Crimean Congo haemorrhagic fever (CCHFV), Zika (ZIKV), and Rift Valley fever viruses (RVFV). Malaria parasite infections were investigated using thick blood smear (TBS) and rapid diagnostics tests (RDT) while arbovirus infections were tested by IgM antibody detection (ELISA) and RT-PCR assays. Data analysis of single or concurrent malaria and arbovirus was performed using R software.

**Results:**

A total of 13,845 patients, including 7387 with malaria and 41 with acute arbovirus infections (12 YFV, nine ZIKV, 16 CHIKV, three DENV, and one RVFV) were enrolled. Among the arbovirus-infected patients, 48.7 % (20/41) were co-infected with malaria parasites at the following frequencies: CHIKV 18.7 % (3/16), YFV 58.3 % (7/12), ZIKV 88.9 % (8/9), DENV 33.3 % (1/3), and RVF 100 % (1/1). Fever ≥40 °C was the only sign or symptom significantly associated with dual malaria parasite/arbovirus infection.

**Conclusions:**

Concurrent malaria parasite and arbovirus infections were detected in the Kedougou region from 2009 to 2013 and need to be further documented, including among asymptomatic individuals, to assess its epidemiological and clinical impact.

## Background

Arboviral infections and malaria are acute vector-borne diseases and concurrent infections are observed [1, 2], especially for dengue in American and Asian tropical regions where their endemic areas overlap extensively [3–10]. However, in African tropical regions, arboviral and malaria parasite co-infections are scarce in the scientific literature and likely under-reported due to the limited number of laboratories capable of diagnosing arboviral infections. Arboviruses are not systematically investigated and are generally only considered by clinicians, at best, when samples test negative for malaria. In addition, arboviral infections are often misdiagnosed as malaria due to their similar clinical presentation [11]. Consequently, this may result in the slow identification of an arboviral disease outbreak and potentially high morbidity and mortality [12–14]. Arboviral and malaria parasite co-infections have previously been reported in Nigeria [11], Senegal [15] and in European travellers in Senegal, Guinea and Sierra Leone [16].

In Senegal, the introduction of malaria rapid diagnostics tests (RDT) in 2007 showed that the prevalence of malaria among acute febrile illnesses (AFI) was largely overestimated while other infections, such as bacteria and arbovirus illnesses were under-reported [17, 18]. In 2009, more robust surveillance of AFI was implemented in Kedougou to detect arboviral infection outbreaks and malaria in order to accurately measure disease morbidity and mortality in this geographical location. In this paper, malaria prevalence and diagnostics as well as co-infections with dengue (DENV), chikungunya (CHIKV), zika (ZIKV), yellow fever (YFV), and Rift Valley fever viruses (RVFV) are reported from 2009 to 2013 in Kedougou region, Senegal, an area known to be endemic for many arboviruses.

## Methods

### Study location

**Fig. 1:**
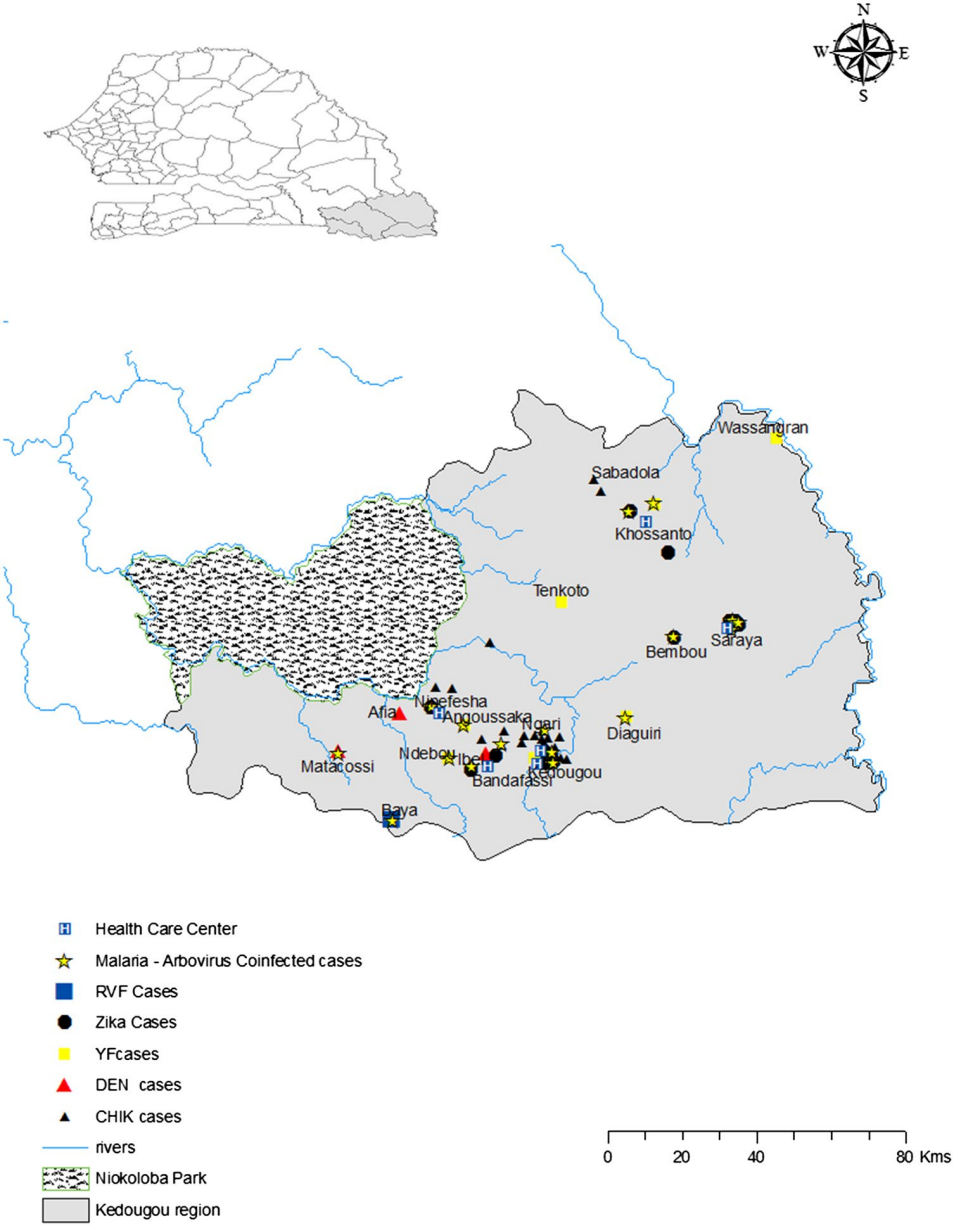
Geographical distribution of arboviruses and malaria/arboviral co-infections in Kedougou. *Green dots* represent confirmed ZIKV infections. *Blue and black triangles* indicate RVF and CHIKV confirmed cases, respectively. *Red dots* represent YFV cases. *Black stars* indicate DENV cases. *Small yellow dots* represent co-infected patients

The study was conducted in the Kedougou region, southeastern Senegal (Fig. 1) with an estimated population of 141,226 inhabitants, among whom 55 % are under 20 years old, and an average density of eight persons per sq km [19]. The Kedougou region borders Guinea, Mali and The Gambia, between isohyets 1200 mm and 1300 mm rainfall per year. The climate is Sudano-Guinean with a single rainy season from May to November [20]. The landscape consists of wooded grassland or woodland and dense gallery forest. The fauna is diverse with herbivores, insectivores, rodents, and monkeys.

### Population and study design

Patients presenting with AFI were recruited from seven healthcare facilities of the Kedougou region, including Ninefesha rural hospital, Kedougou and Saraya health centres, Bandafassi and Khossanto health posts, the Kedougou military health post, and the Catholic Mission mobile team, which targets populations in remote areas (Fig. 1). AFI was defined as any patient older than 1 year of age with a fever (axillar temperature >38 °C) lasting for less than 2 weeks and exhibiting two or more of the following signs or symptoms: headache, myalgia, eye pain, arthralgia, cough, nausea/vomiting, diarrhoea, jaundice, bleeding, and neurological signs. Clinical manifestations and sociodemographic data were recorded on a standardized interview form. From each consented and eligible patient, health workers sampled 5 ml of venous blood in healthcare centres. The same day, samples were centrifuged and serum aliquoted and transported to the field where they were tested and stored at −20 °C until further use.

### Laboratory methods

Human sera were systematically tested for malaria parasite and arbovirus infections. The malaria diagnosis procedure was performed using thick blood smear (TBS) and an RDT (Malaria Antigen P.f, Standard diagnostics, Ingbert, Germany) as previously described [21, 22], while IgM ELISA, real-time RT-PCR and other viral infection detection methods [23–26] were used for detection of DENV, CHIKV, ZIKV, Crimean-Congo haemorrhagic fever (CCHFV) or West Nile virus (WNV), YFV, and RVFV infections. Negative and positive controls were used for serology and molecular methods and any samples with Ct value 35 was considered as positive for RT-PCR. Consequently, a case of confirmed arboviral infection was defined as any AFI that tested positive by any method used for detection of IgM and/or the genome of ZIKV, DENV, CHIKV, RVFV, YFV, CCHFV, or WNV, while a confirmed malaria case was defined as any AFI testing positive for TBS. Only TBS was used for the definition of confirmed malaria case as it is the gold standard for malaria diagnostics. Concurrent infection was defined as any AFI confirmed for both arboviral and malaria infections.

### Statistical analysis of data

Data were analysed using STATA [27] and R software [28]. For small numbers of samples, exact methods, such as exact logistic regression and Fisher’s exact test or other non-parametric methods such as Kruskal–Wallis test, were used for qualitative and quantitative variables to investigate differences in measures between sub-groups. Significance was assigned at p < 0.05 and when pair-wise comparisons were performed among more than two groups, p values corrected for multiple testing by the Bonferroni method were considered.

### Ethics, consent and permissions

The study objectives, benefits and risks were explained in the French language or local dialects to all participants before inclusion. Written informed consent was obtained from all adult participants and from parents or legal guardians of children. The study was examined and approved by the Senegalese National Health Research Committee.

## Results

### Study population

From July 2009 to March 2013, 13,845 patients with AFI (representing 80 % of the patients with AFI attending the healthcare centres) were enrolled from the following seven health facilities: Ninefesha rural hospital (13.5 %), the Kedougou health centre (13.0 %), Saraya health centre (4.8 %), Bandafassi health post (9.7 %), the Military health post (53.8 %), Khossanto health post (3.6 %), and the Catholic mission mobile team (1.4 %). The median age was 16 years, ranged from 1 to 90 years, and the male/female sex ratio was 1:4. Among enrolled patients, 7387 (53.4 %) were malaria-confirmed cases (median age 13 years (range 1–90); male/female sex ratio 1:35), and 41 (0.3 %) were arboviral-infected individuals i.e., 12 YFV, nine ZIKV, one RVF, 16 CHIKV, and three DENV (median age 22 years [range 2–46]; male/female sex ratio 1:4). No patients tested positive for WNV or CCHF. The distribution of positivity for IgM and RT-PCR are summarized in Table 1.

**Table 1:** Arboviruses detected in Kedougou region and test used

Arboviruses	Virological test	
	IgM	RT-PCR
YFV	11	2
CHIKV	4	12
ZIKV	9	0
DENV	3	0
RVF	0	1
Total	27	14

### Co-infection with malaria parasites and arboviruses

**Fig. 2:**
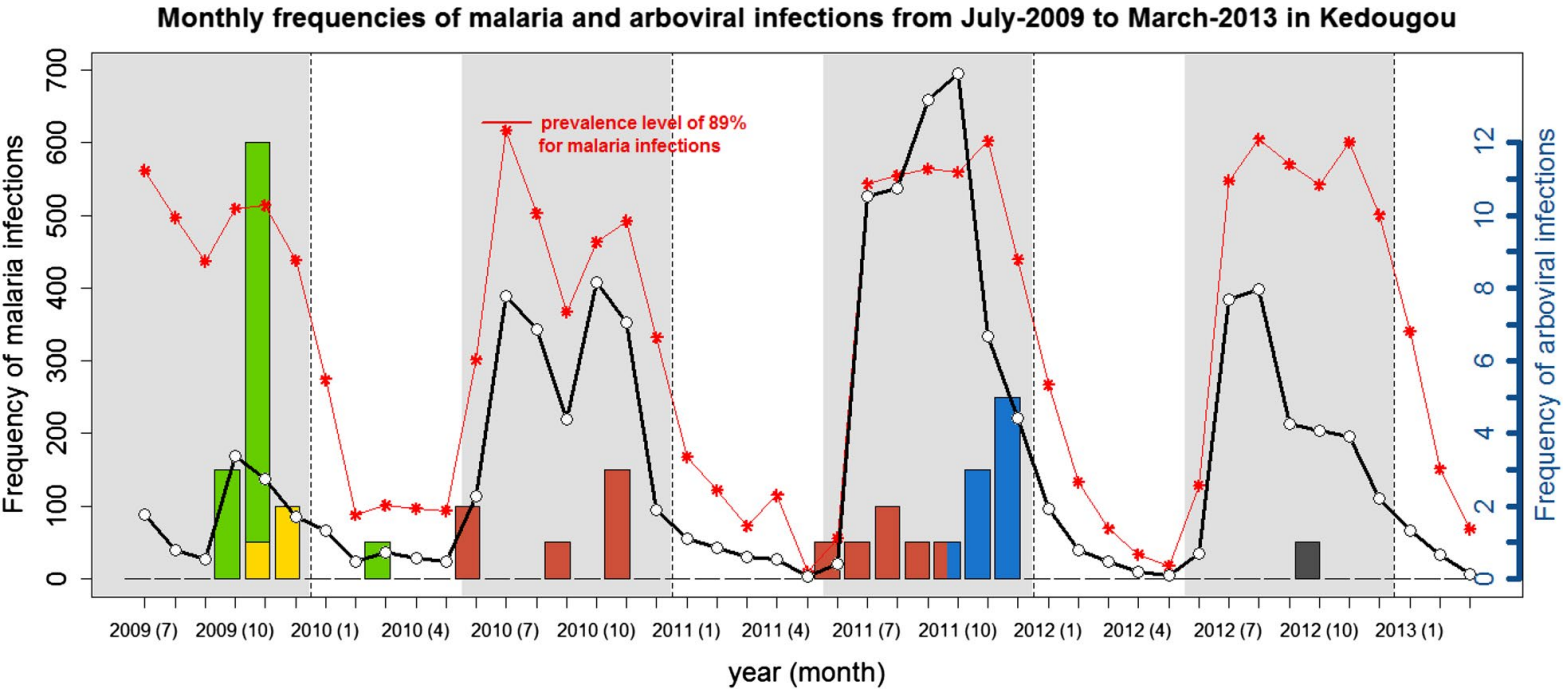
Prevalence of malaria and arboviral infections from 2009 to 2013 in Kedougou. The monthly evolution of malaria infections frequency and prevalence as well as the frequency of arboviral infections from July 2009 to March 2013 in Kedougou. The *solid black line* with *white dots* represents the frequency of blood samples positive for malaria parasites; measures are given by the left-hand y-axis. The *dotted red line* with *red asterisks* corresponds to the ratio ‘number of samples positive to malaria’ divided by ‘total number of samples’. *Vertical bars* represent the frequencies of blood samples positive for arboviruses: *red bars* for YFV, *blue bars* for ZIKV, *green bars* for CHIKV, *yellow bars* for DENV-2, and *dark grey bar* for RVFV. Measures are indicated by the right-hand y-axis. *Vertical dashed lines* separate the plot by year and *grey-shaded zones* correspond to rainy seasons

As shown in Fig. 2, several major patterns can be evidenced from data collected during the 2009–2013 period: (i) the malaria parasite and arboviral infection peaks were coincidental; (ii) the yearly prevalence of malaria was stable with respect to dry and rainy seasons as shown by the red curve; and, (iii) different arboviruses circulated during malaria occurrence in this region of Africa. During rainy seasons, periods of high malaria prevalence, 15 positive IgM for CHIKV (green bars) and three for DENV (yellow bars) in 2009, six for YFV (red bars) in 2010, six for YFV (red bars) and nine for ZIKV (blue bars) in 2011, and one RVFV (dark grey bars) in 2012, were detected among samples. No arboviral infection was observed during dry seasons from 2009 to 2012 except one CHIKV acute illness IgM positive in 2010.

Among patients infected with arboviruses, 48.7 % (20/41) were concurrently harbouring positive testing for malaria parasites. Distinct differences in co-infection rates were observed among the tested arboviruses. Malaria co-infection was identified in 18.7 % (3/16) of CHIKV, 58.3 % (7/12) of YFV, 88.9 % (8/9) of ZIKV, 33.3 % (1/3) of DENV, and 100 % (1/1) RVF patients. These co-infection rates were not significantly different from one another except between CHIKV and ZIKV (Fisher exact p value = 0.021 after multiple tests correction). Given the prevalence of malaria and the circulation of distinct arboviruses in this region, these observed co-infection rates reached expected levels, with the exception of CHIKV where co-infection was significantly lower than expected (empirical p = 0.0089). Patients concurrently infected with malaria parasites and arboviruses were significantly younger, with median age of 14 years compared to 29 years in non-co-infected patients (p = 0.004, Kruskal–Wallis rank test). Among co-infected patients, 67 % presented high grade fever (temperature ≥40 °C), while only ten and 34 % of patients, respectively, were diagnosed with a single arboviral or malaria infection with temperature ≥40 °C (Tables 1 and 2). Therefore, co-infected patients had a significantly higher risk of exhibiting a high-grade fever than those infected with a single pathogen (OR = 19.5, CI = [3.38; 112.45], exact p value = 2 × 10–4). Also any other symptom was found significantly associated with co-infected patients (Table 3).

**Table 2:** Risk of co-infection according to temperature and age

	Coinfected patients		
	OR	95 % CI	p value
Temperature ≥40	12.2	[2–137.7]	2.6 × 10^−3^
Age <15 years	2.4	[0.4–15.6]	0.4

Number of observations: 41; Model score: 12; Pr ≥ score: 1.4 × 10^−3^

## Discussion

This study revealed that concurrent infections of malaria parasites and arboviruses were detected among 48.7 % patients infected with arboviruses in southeastern Senegal. ZIKV was the most prevalent arbovirus in the co-infections with malaria (88.9 %). Further, high-grade fever (≥40 °C) was significantly associated with patients exhibiting dual infection of malaria parasites and arbovirus compared to patients with single malaria attack. Although *Plasmodiumfalciparum, P. ovale, P. malariae,* and *P. vivax* [29] were reported in Senegal, only *P. falciparum* was detected in this study.

Arboviral infections and malaria are known to be endemic in the Kedougou region [13, 14], allowing the occurrence of concurrent infections in patients as previously reported in endemic areas [2]. The overall arboviral and malaria co-infection rates were similar to those reported in previous studies in Nigeria and French Guiana [1, 3, 11]. Evidence of co-infection in ZIKV and malaria parasites is reported. The latter high co-infection rate, rising up to 89 %, may be explained by the almost permanent circulation of ZIKV repeatedly isolated from mosquitoes in the Kedougou region since 1968 [30]. Conversely, the other arboviruses investigated are known to emerge periodically after a few years of silent circulation or absence [31].

An individual can become co-infected when bitten by a mosquito harbouring both the malaria parasite and an arbovirus. In fact, malaria vectors have previously been found infected with arboviruses, emphasizing the plausibility of the dual infection of malaria parasites and an arbovirus within the same mosquito. For example, wild caught *Anopheles funestus,* a major malaria vector in southeastern Senegal and elsewhere in Africa [32], have been found infected with CHIKV and YFV in the Kedougou region ([33], Diallo et al. unpublished data). Moreover, *A. coustani*, which were found infected with CHIKV, YFV and ZIKV in Kedougou region ([30, 33], Diallo et al. unpublished data), may be competent in malaria transmission in this region considering it is abundant, highly anthropophilic and has already been incriminated as a secondary malaria vector in Kenya [34]. Therefore, arbovirus and *Plasmodium* spp co-infection of *A. funestus* and *A. coustani* is possible in nature and needs to be further investigated.

Another mechanism by which a patient may become infected by both *Plasmodium spp* and arboviruses is consecutive bites from two different infected mosquitoes or species (e.g., anopheline vectors for malaria and *Aedes* spp. for arboviruses). When considering the high number of asymptomatic malaria and arboviral infections in endemic regions where both vectors co-exist [1, 35, 36], as Kedougou region, concurrent infections are very plausible through consecutive bites of humans by two infected mosquitoes. The latter condition could even lead to more severe presentations as shown in a previous study concerning DENV and malaria co-infection [36].

Given the similar clinical presentation of arboviral infections and malaria, and the lack of pathognomonic signs and symptoms for any of the diseases, it is difficult to determine which pathogen was responsible for the clinical signs and symptoms in the concurrent infections. For instance, all patients dually infected with malaria parasites and arboviruses had relatively mild and/or non-specific syndromes, including fever, headache, myalgia, body pain, and vomiting. In addition, the fact that arboviral infections are considered by healthcare workers only if malaria tests are negative, sets the stage for the misdiagnosis and under-reporting of concurrent infections. A significant number of the co-infected patients in this study exhibited a fever ≥40 °C compared to patients with malaria or arboviral (Table 3) infection alone. This suggests that high-grade fever could be considered as a differential diagnostic criterion in Kedougou, to trigger further testing for malaria/arbovirus dual infection, as previously suggested for malaria-dengue co-infection [2, 36]. However, given the small number of arbovirus infection detected in this study, further investigation is needed to confirm this observation.

**Table 3:** Main clinical characteristics of co-infected patients and patients infected with only malaria parasites

Symptoms	Arbovirus-infected patients n (%)	Co-infected patients n (%)	p value
Headache	16 (76)	19 (95)	0.18
Eye pain	00 (00)	02 (10)	0.23
Myalgia	12 (57)	06 (30)	0.12
Arthralgia	13 (62)	06 (30)	0.06
Rash	01 (05)	00 (00)	1.00
Vomiting	07 (33)	10 (50)	0.35
Diarrhoea	01 (05)	04 (20)	0.18
Chills	11 (52)	11 (55)	1.00
Cough	07 (33)	04 (20)	0.48

*n* number of patients

One the limitation of the present study is that all the enrolled individuals were febrile patients. However, given that malaria parasites as well as arboviruses can be detected using molecular tools [37] in asymptomatic individuals, studies enrolling asymptomatic individuals are needed to evaluate the real burden of co-infections of malaria and arboviruses in Kedougou area and should be performed in the future.

## Conclusion

This study showed that co-infections between *Plasmodium* spp. and arboviruses are frequent in Kedougou where competent vectors of both diseases are abundant. Vector competence and co-infection of certain malaria vectors, also regularly found infected by arboviruses, deserves further investigation. The frequent detection of arboviral disease outbreaks in the Kedougou region highlights the need to strengthen surveillance of AFI for a better estimation of human impact of arboviruses, as well as morbidity and mortality associated with concurrent malaria and arboviral infections. Finally, the high-grade fever ≥40 °C suggests the possibility of malaria and arboviral infection and should help to establish prompt and better care of individuals.

## Abbreviations

YFV: yellow fever virus
ZIKV: zika virus
CHIKV: chikungunya virus
DENV: dengue virus
TBS: thick blood smear
RDT: rapid diagnostic test
AFI: acute febrile illnesses
ELISA: enzyme-linked immunosorbent assay
RT-PCR: reverse transcription polymerase chain reaction
CI: confidence interval
HRP2: histidine rich protein
WNV: west nile virus
RVFV: rift valley fever viruses
